# Effect of Surrounding Blur on Foveal Visibility

**DOI:** 10.2174/1874364100701010004

**Published:** 2007-11-07

**Authors:** Hiroyuki Sakai, Takayuki Kannon, Shiro Usui

**Affiliations:** 1 Laboratory for Neuroinformatics, RIKEN Brain Science Institute, Japan; 2 Department of Information and Computer Sciences, Toyohashi University of Technology, Japan

## Abstract

Visibility of a simple stimulus is known to be determined not only by its physical contrast, but also by the configuration of surrounding stimuli. In this study, we investigated the surrounding modulation of foveal visibility of a blurred target. Subjects were instructed to respond to the gap orientation of a Gaussian-blurred Landolt ring presented at a fixation point with a surrounding stimulus. The correct response rate was measured as a metric of the foveal visibility. Results were subsequently compared among different surrounding stimulus conditions. Results showed an improvement in the subjects’ performance when low-pass white noise filtered with the same Gaussian function used for the target was presented in the surrounding area, although no effect was observed using high-contrast white noise. A performance improvement was observed when the surround stimulus had an intermediate contrast in the spatial frequency band necessary for identifying the target orientation.

## INTRODUCTION

Retinal images are two-dimensional perspective projections of three-dimensional real space viewed through the eye’s optics. Therefore, when the focal length is adjusted to view an object of interest, the surrounding objects outside the depth of focus create blurry images on the retina. Even the retinal image of the object of interest becomes blurry because of accommodative errors and higher-order aberrations. For those reasons, blur is an intrinsic feature of retinal images.

Previous studies [1–5] have demonstrated that a certain period of exposure to the defocus blur (blur adaptation) improves visual acuity, suggesting the presence of a neural circuit that compensates for blur. More recently, Artal *et al*. [6] suggested that the mechanism underlying blur compensation functions effectively for more complex blur caused by higher-order aberrations. In addition, blur adaptation reportedly affects perceptual blur sensitivity [5] and judgment of focus [7, 8].

In contrast to blur adaptation, little information is available related to the effects of surrounding blur. In the current study, we examined an effect of a blurred stimulus in the surrounding area on the foveal visibility of a blurred target. As a metric of foveal visibility, we used the correct response rates in a simple visual acuity task using Gaussian-blurred Landolt rings presented with various surround stimuli. The results were then compared among different surround-stimulus conditions.

## EXPERIMENT 1

### Subjects

Two authors, HS and TK, participated in this experiment along with two subjects, NS and NY, who were naïve to the purpose of the study. All participants had normal or corrected-to-normal vision. The participants’ ages were 21–29 years old. The experimental procedures conformed to the tenets of the Declaration of Helsinki. Informed consent, the criteria for which were approved by our institutional review board, was obtained after the experimental protocol was explained to the participants.

### Apparatus

Stimuli were produced using a personal computer with a video card (VSG 2/5; Cambridge Research Systems, Ltd.) System and displayed on a monitor (FlexScan T962; EIZO Nanao Corp.) in a dimly lit room. The monitor had a resolution of 1024 × 768 pixels with a refresh rate of 80 Hz; it was calibrated using a photometer (LS100; Konica Minolta Holdings Inc.). The stimuli were viewed binocularly at a viewing distance of 1.5 m.

### Stimuli

**Fig. (1):**
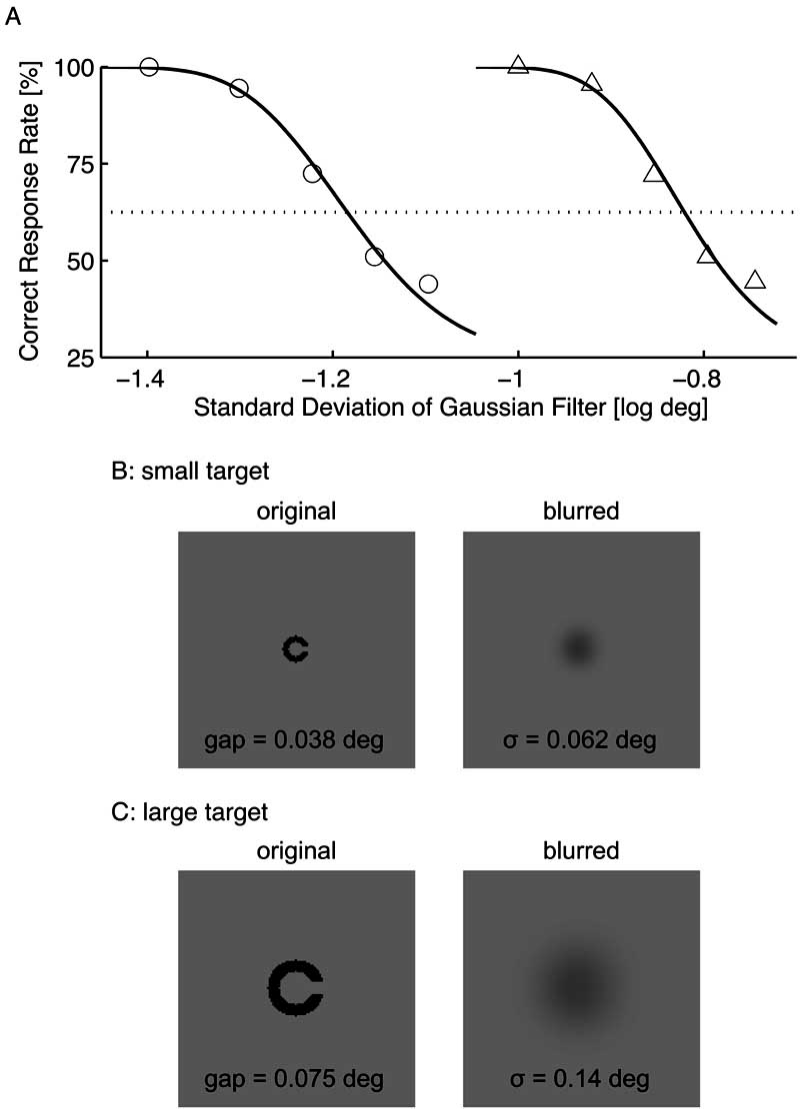
Result from the preliminary experiment to determine the Gaussian filter sizes. The standard deviation that resulted in a correct response rate of 62.5% was determined using constant stimuli **(A)**. For this subject (TK), the filter sizes were 0.062 deg for small target (circles) and 0.14 deg for large target (triangles). The lower panels show examples of the small **(B)** and large **(C)** targets, whose correct response is rightward.

Each stimulus comprised a centered target and surrounding noise. The target was a blurred Landolt ring. The Landolt ring was drawn as a dark line (5 cd/m^2^) on a homogeneous background (30 cd/m^2^) and then blurred using a two-dimensional Gaussian function,

gx,y=12πσ2exp−x2+y22σ2

where (*x, y*) indicates position, and *σ* is a standard deviation that is referred as the filter size. Two target sizes were used in this experiment; the small and large targets had respective gap sizes of 0.038 deg (LogMAR 0.36) and 0.075 deg (LogMAR 0.65) (Figs. **1B** and **1C**). The line width was equal to the gap size in each target. The filter size was determined using constant stimuli for each target size and subject so that the subject achieved a correct response rate of 62.5% (an intermediate level of performance between random chance and a perfect performance). Fig. **1A** shows an example of the preliminary results. For this subject (TK), the filter sizes were 0.062 deg for the small target (Fig. **1B**) and 0.14 deg for the large target (Fig. **1C**). The filter size of the small target (*σ_s_*) was approximately half that of the large target (*σ_l_*) for all subjects (*σ_s_* = 0.062 ± 0.003 deg vs. *σ_l_* = 0.14 ± 0.01 deg).

The surround stimuli were annular disks with internal and external diameters of 1 and 9 deg. The target stimulus was embedded in a 1-deg central circular region. Four surround stimuli were used in this experiment: white noise (0.95 contrast pseudorandom binary noise), two low-pass white noises generated with the same filters as those used for the targets (*σ_s_*-noise and *σ_l_*-noise), and homogeneous gray as a control condition. For all stimuli, the average luminance was set to 30 cd/m^2^.

### Procedure

We measured the correct response rate for identifying the gap orientation of the target using a four-alternative forced-choice procedure (upward, downward, rightward, or leftward). After 180 s of adaptation to a homogeneous gray background (30 cd/m^2^), a trial sequence was activated. Each trial consisted of a fixation period of 3 s, followed by a no-fixation period of 0.5 s, a stimulus interval of 1.5 s, and a second no-fixation period of 0.5 s. The subjects were instructed to judge the gap orientation during the fixation period for the next trial. The fixation point was a solid, dark red (10 cd/m^2^) square, which subtended a visual angle of about 2 arcmin. The stimulus was presented so that the center corresponded to the fixation point.

The correct response rate was computed from 300 responses for each surround stimulus and target size. Subjects therefore should have performed more than 1,000 trials for each target size. To avoid fatigue, we divided the trials into 12 blocks and allowed the participants to take a 15-min break after every three blocks. All surround stimuli were presented randomly during each block with an equal probability; different sizes of targets were conducted in different blocks.

## RESULTS & DISCUSSION

**Fig. (2):**
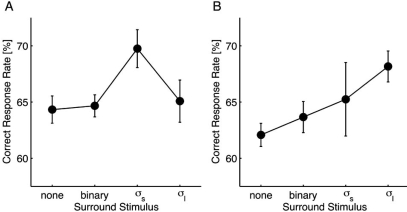
Psychophysical data from Experiment 1. For the small target **(A)**, the correct response rate was significantly improved by surrounding low-pass white noise filtered with the same Gaussian function used for the target (*σ_s_*). In addition, a similar tendency was observed for the large target **(B)**. For both targets, high contrast (0.95) binary noise did not produce an observable effect. Error bars represent the standard error.

The correct response rates from different surround stimulus conditions were compared using one-way repeated measures analysis of variance (ANOVA) followed by paired Dunnet’s post hoc tests. For the small target (Fig. **2A**), the main effect of the surround stimulus was significant [*F*(3, 3) = 4.615; *p *= 0.032]. In comparison to the control condition, *σ_s_*-noise resulted in a significant performance improvement (*p *< 0.05), whereas the other stimuli did not affect the performance (*p *> 0.05). For the large target (Fig. **2B**), although the main effect was unclear [*F*(3, 3) = 3.203; *p *= 0.076], the *σ_l_*-noise resulted in a significant performance improvement compared to the control condition (*p* < 0.05).

These results demonstrate that foveal visibility is improved when the target is surrounded with a certain level of low-pass white noise, suggesting the presence of a deblurring mechanism that depends on the spatial context. We also found that the deblurring effect depends on the target size, which suggests that mechanisms that are selective for spatial frequency are involved in the deblurring process because a spatial frequency band necessary for identifying the gap orientation would be highly correlated with the target size. Relationships between the target and surround stimuli in the spatial frequency domain are described in the next section.

## EXPERIMENT 2

Experiment 1 revealed that the surrounding blur modifies the foveal visibility of a blurred target and the surround modification depends on the target size. Then we ascribed it to different signal bands of the targets. In this experiment, we identify the signal band using the same protocols as those used for Experiment 1.

### Methods

To identify the signal band, we applied rectangular notch filters to the target as follows: after transforming a target to the spatial frequency domain, we zeroed the amplitude spectrum in a specific band (notch filtering), and transformed it back to the spatial domain. The band was set as < 2, 2–4, 4–8, and > 8 cpd (cycle per degree) for the small target, and < 2, 2–4, and > 4 cpd for the larger target. Three subjects (HS, NS, and NY) participated in this experiment. The remaining methods were identical to those described for Experiment 1, provided that no surround stimulus was presented.

### Results & Discussion

**Fig. (3):**
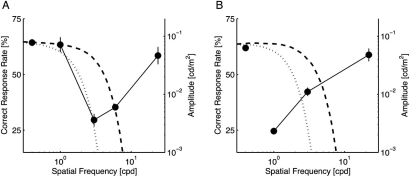
Correct response rates of notch-filtered targets. Each data point was plotted at the center frequency of the applied notch filter except for the leftmost point, which represents the performance level without notch filtering. Error bars show the standard error. For the small target **(A)**, the signal band ranged from approximately 2 to 8 cpd. For the large target **(B)**, it shifted to lower spatial frequencies (less than 4 cpd). In each panel, the amplitude spectra of surrounding stimuli used in Experiment 1 were superimposed (refer to the right ordinate). Dashed and dotted curves respectively represent the *σ_s_*-noise and *σ_l_*-noise.

Fig. (**3**) shows the correct response rate of the participants for each target size. The leftmost point in each panel represents the control condition (no notch filter); the other points are plotted at the center frequency of the applied notch filter. For both targets, one-way repeated measures ANOVA revealed a significant main effect of the notch filter frequency (*p* < 0.0001 for both target sizes). In comparison to the control condition, the performance was significantly degraded, as demonstrated by the lack of bands from 2 to 8 cpd for the small target (*p* < 0.01; Fig. **3A**) and less than 4 cpd for the large target (*p* < 0.01; Fig. **3B**). In addition, no other tested conditions produced an improvement in the participants’ performance.

These results demonstrate that, as expected, the signal band shifts to a lower frequency with increased target size. Interestingly, no performance improvement was found for any of the bands we tested: no clear noise band interferes with the task as a masker. This fact suggests that, if the sensitivity changes in the spatial frequency-selective mechanisms resulted in the performance improvement observed in Experiment 1, the surrounding low-pass white noise facilitates the sensitivities responsible for a certain band overlapping with the signal band. In addition, the sensitivity suppression in the remainder of the band does not explain the performance improvement.

## GENERAL DISCUSSION

We investigated the effect of surrounding blur on the foveal visibility of a blurred target using a visual acuity task with Gaussian-blurred Landolt rings as the target and observed a significant improvement in the participants’ performance when low-pass white noise that had been filtered with the same Gaussian function as that used for the target was presented in the surrounding area.

**Fig. (4):**
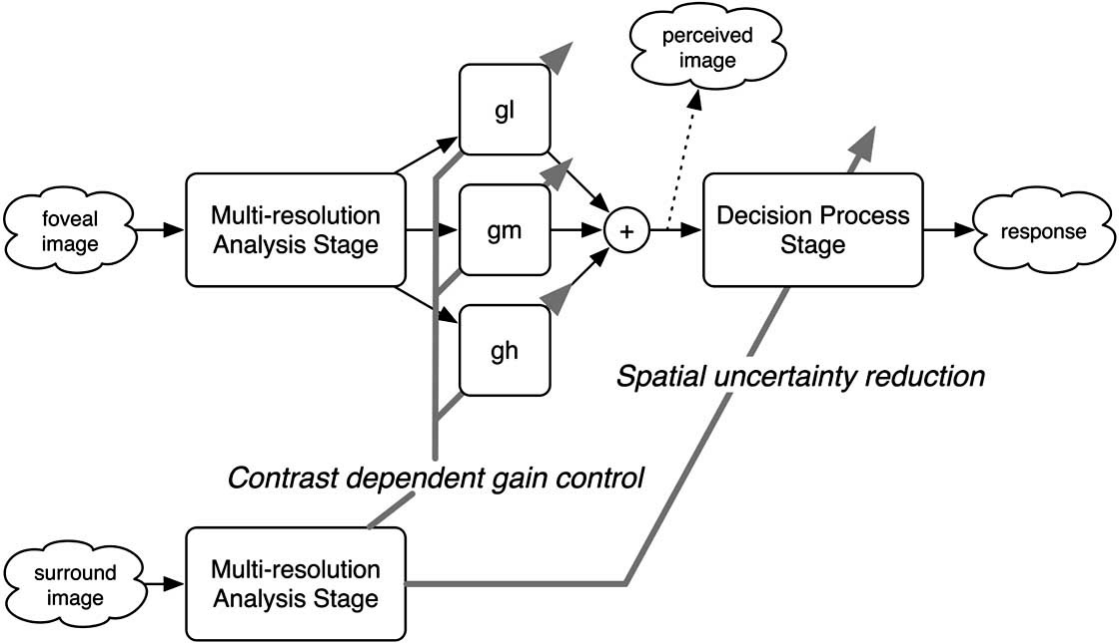
Simple computational scheme for the surround modulation on the foveal visibility of a blurred target (see text for details).

As a framework for the mechanisms underlying the surround modulation of the foveal visibility, we propose a simple computational scheme (Fig. **4**). The scheme assumes two stages in two pathways. The stage consists of units for multiresolution analysis (MRA) and decision process (DP), whereas the pathways are responsible for the foveal and surround regions. In the foveal pathway, the MRA stage decomposes an input foveal image into low, middle, and high spatial frequency components, which corresponds to early visual cortices with receptive fields that are tuned to a specific spatial frequency. During the DP stage, the orientation of a Landolt ring is determined based on the perceived image that is reconstructed as a weighted sum of the outputs of the MRA stage. The surrounding pathway also contains an MRA stage, but not the following DP stage because, in our experiments, the visual acuity task was performed only with the foveal region. In this scheme, there are expected to be two possible types of surround modulation: gain control for the weighted sum and improvement of the DP stage.

A subject is required to attend to a large spatial area because it is difficult to localize the target when a target stimulus is not very visible. According to signal detection theory, such spatial uncertainty is predicted to impair target detection [9]. Conversely, if a surround stimulus reduces the spatial uncertainty about the target location, then the detection performance would improve, which might represent the effect of the surround stimulus during the DP stage. For instance, Petrov *et al*. [10] has recently shown that the contrast detection facilitation by collinear flankers is largely attributable to spatial uncertainty reduction, although this result remains controversial [11–13]. However, it seems unlikely that our result is explainable only by a decrease in the spatial uncertainty because the surrounding *σ_s_*-noise significantly improved the performance of the participants for the small target (Fig. **2A**), but not for the large target (Fig. **2B**). A significant improvement in the performance would also have been observed for the large target if the decreased spatial uncertainty with the surrounding *σ_s_*-noise were a major contributor to our results.

On the other hand, gain control during the MRA stage can explain our results as follows. For simplicity, let *S_s_* and *S_l_*, which denote the signal bands of the small and large targets, match the middle and low frequencies of the MRA stage, respectively. Note in Fig. (**3**) that *S_s_* was higher than *S_l_*. To explain the improvement in the performance shown in Fig. **2A**, the surrounding *σ_s_*-noise would have to increase the gain for the middle frequency *g_M_* selectively and not affect the gain for the low frequency *g_L_*. Similarly, the *σ_l_*-noise would increase *g_L_* rather than *g_M_*. For the small target, if the low amplitude of the surround stimulus with a higher spatial frequency than *S_s_* caused the increase in *g_M_*, the *σ_l_*-noise would improve the performance as well as the *σ_s_*-noise; such, however, was not the case (Fig. **2A**). On the other hand, if the high amplitude of the surround stimulus with a lower spatial frequency than *S_s_* caused an increase in the gain, the *σ_s_*-noise would improve the performance of the participants for the large target; again, this was not observed (Fig. **2B**). Consequently, using a process of elimination, we conclude that the *moderate* amplitude of the surround stimulus around *S_s_* caused the increased gain in foveal vision. In the case of the large target (Fig. **3B**), *S_l_* roughly matched a band with the moderate amplitude of the *σ_l_*-noise, as in the case of the small target.

A substantial amount of psychophysical and electrophysiological data is associated with the surround modulation of spatial frequency-selective mechanisms (for review, see Ref. 14). Several lines of evidence have demonstrated that a surround stimulus with moderate contrast facilitates contrast detection sensitivity for a target stimulus when both stimuli have the same spatial frequency and an orthogonal orientation [11, 12, 15, 16]. This contrast-dependent surround facilitation might underlie the surround modulation on the foveal visibility we observed. However, further studies might be needed to examine whether our results, which were observed using stimuli defined by various spatial frequencies and orientations, are predictable based on the ensemble of evidence that has been obtained using simple stimuli such as grating and Gabor patterns. Solomon and Morgan [17] have shown that additional noncollinear flankers diminish the contrast detection facilitation that results from collinear flankers, although the noncollinear flankers themselves have no significant effect of contrast detection. That fact implies complex mutual interactions among processes for various components of a visual scene. Moreover, McDonald and Tadmor [18] found that the maximum suppression of perceived contrast in foveal vision occurs when the surround stimulus has an amplitude spectrum of a natural scene. This result suggests that the second-order statistics of a surround stimulus also affect the foveal perception.

In addition, according to a recent paper of Rucci *et al*. [19], fixational eye movements can enhance the fine spatial detail of a visual stimulus. Since the stimulus interval of 1.5s in our experiments was enough to elicit the effect [19], it is undeniable that the experimental conditions in which an improvement of the performance was observed triggered appropriate fixational eye movements for the deblurring of retinal images. In fact, subjects reported that a surrounding stimulus was also frequently perceived as sharper when the orientation of a blurred Landolt ring was detected clearly, which suggests the involvement of a mechanism that affects the entire (not local) retinal image. In that regard, the deblurring effect of fixational eye movements might be a dominant candidate.

The above discussions on possible mechanisms underlying the deblurring effect were based on our results obtained under certain experimental conditions. In order to reach a more robust conclusion, further experiments are needed in the future. For instance, more ecological metric of the foveal visibility should be proposed as an alternative to the correct response rate in a visual acuity task. Additionally, it is important to employ natural images as surrounding stimuli. Unlike noise images, natural images have a meaningful phase structure such as edges and textures. The phase structure is expected to give rise to additional blur information, which might enhance the deblurring effect.
